# Anti-Cancer Drugs: Trends and Insights from PubMed Records

**DOI:** 10.3390/pharmaceutics17050610

**Published:** 2025-05-04

**Authors:** Ferdinando Spagnolo, Silvia Brugiapaglia, Martina Perin, Simona Intonti, Claudia Curcio

**Affiliations:** 1 Department of Molecular Biotechnology and Health Sciences, University of Turin, Piazza Nizza 44bis, 10126 Turin, Italy; ferdinando.spagno57@edu.unito.it (F.S.); silvia.brugiapaglia@unito.it (S.B.); martina.perin@unito.it (M.P.); simona.intonti@unito.it (S.I.); 2 School of Advanced Defence Studies, Defence Research & Analysis Institute, Piazza della Rovere 83, 00165 Rome, Italy; 3 Defense Institute for Biomedical Sciences, Via Santo Stefano Rotondo 4, 00184 Rome, Italy

**Keywords:** PubMed, meta-analysis, anti-cancer drugs, combined therapy

## Abstract

**Background:** In recent years, there has been an exponential growth in global anti-cancer drug research, prompting the necessity for comprehensive analyses of publication output and thematic shifts. **Methods**: This study utilized a comprehensive set of PubMed records from 1962 to 2024 and examined growth patterns, content classification, and co-occurrence of key pharmacological and molecular terms. **Results**: Our results highlight an exponential rise in publications, with an annual compound growth rate of over 14%, influenced by advancements in digital knowledge sharing and novel therapeutic breakthroughs. A pronounced surge occurred during the COVID-19 pandemic, suggesting a sustained shift in research dynamics. The content analyses revealed a strong emphasis on classical chemotherapeutic agents—often studied in combination with targeted therapies or immunotherapies—and a growing focus on immune checkpoint inhibitors and vaccine platforms. Furthermore, co-occurrence networks indicated robust links between chemotherapy and supportive care, as well as emerging synergies between immuno-oncology, precision medicine approaches. **Conclusions**: Our study suggests that while novel modalities are reshaping treatment paradigms, chemotherapy remains central, underscoring the value of integrative regimens. This trend toward personalized, combination-based strategies indicates a transformative era in oncology research, where multidimensional data assessment is instrumental in guiding future therapeutic innovations.

## 1. Introduction

Cancer is a leading cause of death worldwide, driving ongoing research into anti-cancer drugs and treatment advancements [1]. Over recent decades, therapeutic strategies have evolved from traditional cytotoxic agents like platinum-based compounds and taxanes [2] to targeted therapies that inhibit molecular drivers (e.g., EGFR, HER2, BRAF) [3,4] and cancer vaccines [5]. Immunotherapy, especially immune checkpoint inhibitors (ICIs) targeting CTLA-4 and PD-1/PD-L1, has revolutionized treatment by using the immune system to achieve durable remissions.

Despite these advancements, chemotherapy remains essential, especially in resource-limited settings and as part of combination regimens. Drug resistance, driven by tumor cell mechanisms like ATP-binding cassette transporters and altered apoptotic pathways, remains a significant challenge. As a result, research focuses on overcoming chemoresistance, improving drug delivery, and personalizing treatments with biomarkers.

Despite these advances, classical chemotherapy remains essential, especially in low-resource settings and as a base in combination therapies [6].

Research is intensifying on overcoming chemoresistance, improving targeted delivery, and using biomarkers to personalize treatment [6]. Preventive vaccines for human papillomavirus (HPV) and hepatitis B virus (HBV) have shown success in reducing infection-related cancers, while therapeutic vaccines—peptide-based, dendritic cell, and mRNA—are being developed to stimulate tumor-specific T-cell responses [7,8,9,10,11,12].

Combining ICIs with oncolytic viruses or cancer vaccines offers promise for overcoming resistance [13,14]. Advanced delivery systems like liposomes and nanoparticles enhance chemotherapy’s effectiveness and reduce toxicity [7]. These strategies reflect a shift toward precision oncology, integrating immunotherapy, genomics, and personalized care to improve outcomes.

This study uses PubMed data from 1962 to 2024 to analyze trends in cancer drug development through bibliometric and semantic methods. It highlights the rise in targeted and immune-based therapies alongside chemotherapy, revealing key challenges like resistance and side effects, and providing a framework to guide clinical decision-making.

## 2. Materials and Methods

We retrieved PubMed records from 1962 to early 2025 using the ENTREZ API, targeting articles that included keywords and substances related to anti-cancer drugs.

To quantify the number of articles published, the Compound Annual Growth Rate (CAGR) was calculated, which measures the constant growth rate over time using the following formula:

(1) $$CAGR={\left(\frac{V_{f}}{V_{i}}\right)}^{\frac{1}{n}}-1$$

where Vf = 3497 (publications in 2024); Vi = 1 (publications in 1962); and n = 62 years (from 1962 to 2024). This method was selected for its ability to estimate average annual growth by smoothing out year-to-year fluctuations, thereby offering a reliable representation of overarching trends, even in the presence of occasional inconsistencies within the dataset.

After collecting the full XML metadata for each PMID, we processed the data in R (version 4.4) to perform bibliometric, textual, and classification analyses. Our scripts parsed all records, transforming them into structured tables for further exploration. Publication counts were then aggregated by year, country of origin, and number of authors, enabling trend analyses and descriptive statistics. For content assessment, we grouped relevant terms into categories (e.g., chemotherapy agents, immune checkpoints, targeted therapies) to visualize their temporal evolution. We also extracted trigrams from the *NameOfSubstance* field to capture common three-term co-occurrences, normalized the data based on each trigram’s maximum recurrence, and examined shifts in publication focus before and during the COVID-19 pandemic. All intermediate tables, scripts, and summaries are available upon request or in the Supplementary Materials.

The classification of terms was determined through a consensus-based methodology. Initially, all trigrams derived from the *NameOfSubstance* field were identified and ranked. Subsequently, the top 250 distinct trigrams were selected and each individual *NameOfSubstance* was assigned to the most appropriate class based on the authors’ consensus.

**Figure 1:**
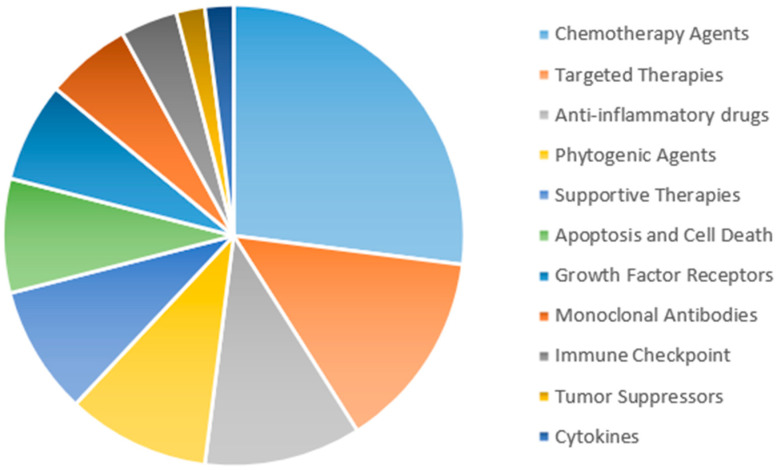
A pie chart representation of the *NameOfSubstance* distribution by category, illustrating the classification of substances related to anti-cancer drug research. Each colored section corresponds to a distinct category and the size of each section is proportional to the distribution of the corresponding terms.

Eleven anti-cancer classes were identified:

1. **Apoptosis and Cell Death**: Proteins and mechanisms involved in programmed cell death (e.g., caspases, Bcl-2, p53), crucial in tumorigenesis and cancer treatment.
2. **Chemotherapy Agents**: Cytotoxic drugs that damage DNA, inhibit nucleotide synthesis, or interfere with cell division (e.g., alkylating agents, antimetabolites, platinum compounds).
3. **Growth Factor Receptors**: Characterized primarily by their direct modulation of oncogenic signaling pathways controlling angiogenesis (e.g., VEGF/VEGFR-2/KDR), cell proliferation, differentiation, and survival (e.g., EGFR, ERBB2/HER2, IGF-I, FGF, TGF-β).
4. **Immune Checkpoint**: Defined specifically by their immunomodulatory action, reversing tumor-induced immune suppression through the targeted inhibition of checkpoint molecules (e.g., PD-1/PD-L1 axis, CTLA-4), thus restoring antitumor T-cell activity and significantly improving patient outcomes in various tumor types.
5. **Cytokines**: Immune-modulating cytokines (e.g., interferons, interleukins) used as adjuvants to enhance immune response against cancer.
6. **Anti-inflammatory Drugs**: NSAIDs and COX inhibitors (e.g., aspirin, celecoxib) with potential roles in cancer prevention and treatment through inflammation modulation.
7. **Monoclonal Antibodies**: Distinguished by their high antigen specificity, representing a therapeutic platform engineered to selectively bind and neutralize defined tumor-associated antigens across diverse malignancies (e.g., CD20, CD19) and advanced antibody formats (e.g., immunoconjugates, immunotoxins, bispecific antibodies).
8. **Phytogenic Agents**: Plant-derived compounds (e.g., alkaloids, flavonoids) with anti-cancer potential.
9. **Supportive Therapies**: Non-anti-cancer drugs (e.g., analgesics, antiemetics) that manage cancer-related symptoms and improve patient quality of life.
10. **Targeted Therapies**: Molecules targeting key signaling pathways (e.g., tyrosine kinase inhibitors, mTOR inhibitors), offering more precise treatments than chemotherapy.
11. **Tumor Suppressors**: Proteins (e.g., p53, PTEN) that regulate cell growth, often inactivated in cancer, with research focused on reactivating or targeting these pathways.
  Detailed information on the topics in each class is available in Appendix A.1, and an overview of the drugs is shown in Figure 1.

**Figure 2:**
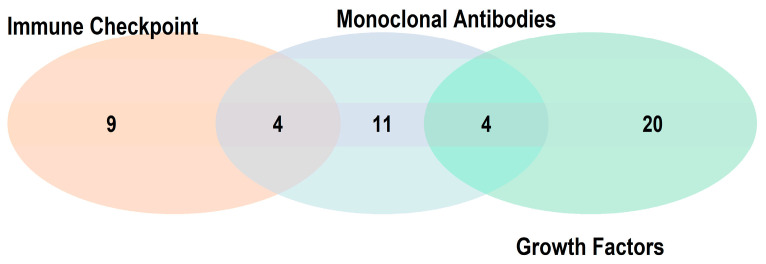
A Venn diagram depicting potential overlaps among the three identified classes (“Growth Factors,” “Immune Checkpoint,” and “Monoclonal Antibodies”), considering only terms counting more than 50 PMIDs. The intersection between *Growth Factors* and *Monoclonal Antibodies* includes trastuzumab, cetuximab, panitumumab, and bevacizumab. The intersection between *Immune Checkpoint* and *Monoclonal Antibodies* includes nivolumab, pembrolizumab, ipilimumab, and atezolizumab. No biologically significant overlap was identified between *Growth Factors* and *Immune Checkpoint*, nor among all three categories simultaneously.

Although “monoclonal antibodies” represent a broad and structurally diverse therapeutic category—including agents targeting both growth factor receptors and immune checkpoint molecules—we identified distinct research trends that justify treating ICIs as a separate class. Given the clinical significance and mechanistic specificity of therapies targeting growth factor pathways and immune checkpoints, we found it scientifically appropriate to categorize these independently. As a result, we limited the “monoclonal antibodies” category to those targeting well-defined tumor-associated antigens with established clinical applications. This distinction reflects both structural and functional differences. Specifically, agents in the “Growth Factor Receptor” category target key signaling pathways involved in angiogenesis and tumor cell proliferation, while “Immune Checkpoint” therapies are defined by their capacity to restore suppressed immune responses, leading to improved outcomes across a wide range of cancers. Figure 2 illustrates a Venn diagram depicting the potential overlaps among the three classes of terms, each of which includes compounds associated with more than 50 PMIDs, with detailed list provided in the Supplementary Materials: file ImmuneCheckpoint_filtered_immune_data_with_titles.tsv).

## 3. Results

### 3.1. General Trend in Scientific Production (1962–2024)

**Figure 3:**
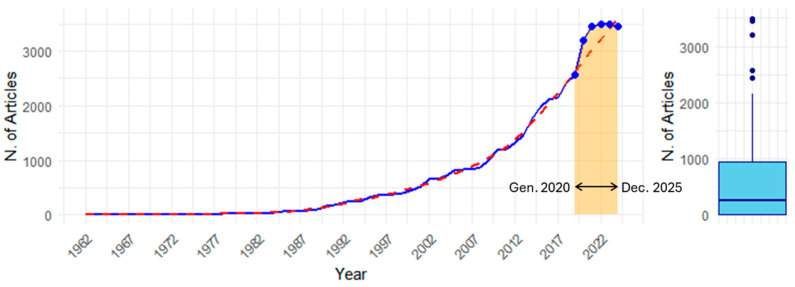
A time series of article counts and their distribution. The left line plot depicts the growth in the number of publications over the years, while the right boxplot shows the interquartile range, with the central line indicating the median, whiskers representing the data range, and points above representing outliers.

The analyzed dataset shows a right-skewed distribution with significant variability in scientific publications from 1962 to 2024. The mean number of publications is 733.51, but the median is much lower at 245, indicating that most years had fewer publications, with occasional high peaks (Figure 3, Table 1). The data dispersion (1037.40 ± 3496) and interquartile range (933.5) reflect a large difference between the first and third quartiles. A skewness index of 1.52 confirms a long right tail, and a kurtosis near 1 suggests a slightly flatter distribution compared to a normal one. This variability is likely driven by factors such as technological advancements, regulatory changes, or specific events that caused spikes in research activity.

The number of scientific publications has grown exponentially, with a notable rise in recent decades. From just one article in 1962, publications reached 3497 in 2024, with a Compound Annual Growth Rate (CAGR) of 14.05%. This growth is driven by factors such as increased global research investment, expanded digital access, greater international collaboration, open-access journals, and advancements in scientific technology.

### 3.2. Geographical Scientific Contribution in Publication

**Figure 4:**
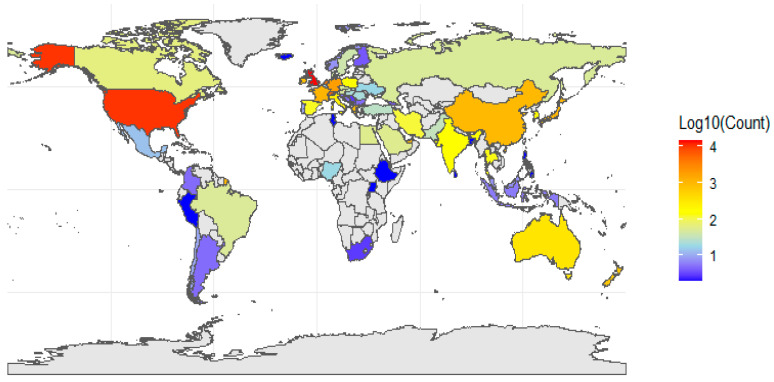
Geographical distribution of total published articles by editor countries, displayed on logarithmic scale to enhance visualization.

The distribution of scientific publications shows a global disparity, with a few countries dominating research output (Figure 4). Developed nations, particularly the United Kingdom (14,605 publications), United States (10,677), and Switzerland (5581), lead in scientific publications. Other countries like the Netherlands (4501) and Germany (1895) also contribute significantly. In contrast, many countries publish fewer than 50 articles (Figure 4).

The number of authors per article reflects research complexity and collaboration levels. From 1969 to 2024, the median number of authors typically ranged from 2 to 7, with most articles involving fewer than 10 authors (Appendix A.2). The interquartile range shows smaller teams dominate publishing. However, after 2015, there was an increase in outliers (articles with over 50 authors), coinciding with large-scale global initiatives, particularly following the OMICs revolution [15,16,17].

### 3.3. Anti-Cancer Drug Classification and Grouped Analysis

To enhance the analysis of anti-cancer drug research, we focused on the *NameOfSubstance* field in PubMed records. By analyzing these fields, we identified key relationships and trends in the research area. The analysis was conducted at three levels, single-element, two-element, and three-element groups, enabling us to achieve the following objectives:

- *Quantitative tracking:* Measured the frequency of cited elements, revealing their temporal evolution and role in shaping scientific discourse.
- *Link analysis:* Examined relationships between elements, uncovering dynamics within and across research themes.
- *Article classification:* Categorized articles into general topics and niche areas, providing insight into the research landscape.

This approach revealed both macro- and micro-level trends, helping us understand the thematic structure of anti-cancer drug research and the evolution of scientific ideas.

**Figure 5:**
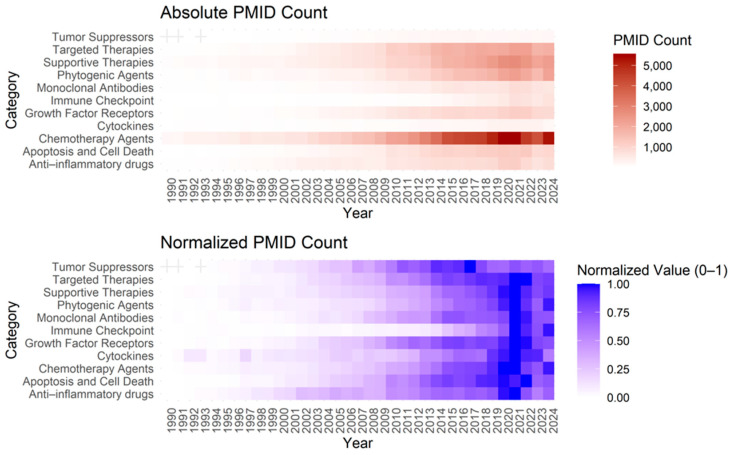
Heatmaps illustrating the temporal evolution of PMID counts across different categories of anti-cancer drug research. Top Panel (Absolute PMID Count): Raw number of PubMed-indexed publications per category from 1990 to 2024. Darker shades indicate a higher volume of research activity. Bottom Panel (Normalized PMID Count): Relative trend within each category, normalized between 0 and 1 for intra-category comparison purposes.

The *NameOfSubstance* field was chosen for its specificity and consistency in identifying pharmacological compounds (Table 2), making it ideal for bibliometric analysis. Unlike other indexing fields, it avoids redundancy and ensures reproducibility through standardized terms. This enables effective tracking of research trends, emerging drug combinations, and shifts in pharmaceutical focus—particularly in response to major events like **COVID-19**. It also supports hierarchical classification of therapies, enhancing insight into the evolution of treatment categories. Over the past three decades, oncology publications have increased significantly, with recent plateaus suggesting a transition toward more complex, personalized, and combination-based therapies, as shown in Figure 5 and detailed in Appendix A.3.

**Figure 6:**
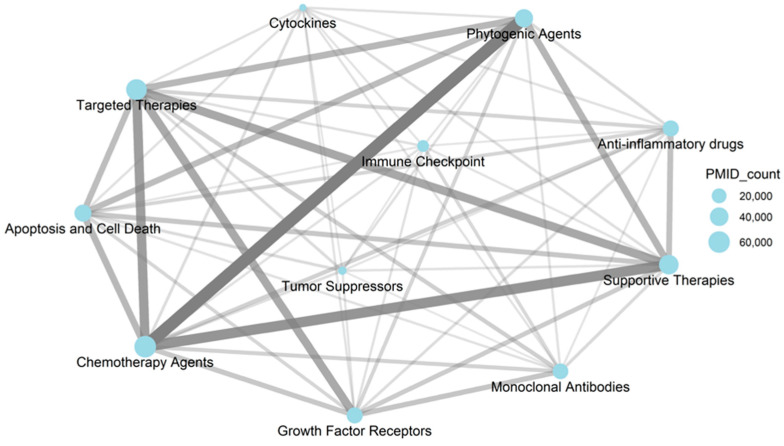
The relationships within the categories. Graph description: Nodes (Categories): Each node represents a category of drug-related research. The size of the node is proportional to the number of PubMed articles (PMID count) associated with that category. Larger nodes indicate categories with a higher number of related articles. Smaller nodes represent categories with fewer associated publications. Edges (Connections): The edges represent the co-occurrence of categories in the same PubMed articles. Thicker and darker edges indicate a stronger relationship (higher number of shared articles). Thinner and lighter edges signify weaker relationships with fewer shared publications.

After classification, a first global assessment was conducted to analyze the relationships within categories by means of an undirected weighted graph, with *Nodes* representing drug-related categories, *Edges* representing the relationships between categories established when two categories co-occur in the same PubMed article, and *Weights* indicating the number of shared PubMed IDs (PMIDs) between two categories, meaning the strength of the association (Figure 6).

**Figure 7:**
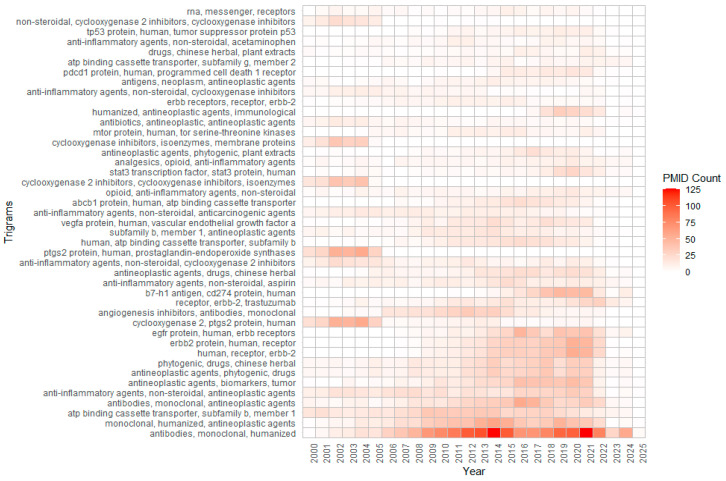
Recurring trigrams of anti-cancer drugs overtime. An interactive graph of all trigrams is available in the Supplementary Materials NameOfSubstance_trigrams_full.html.

Figure 7 illustrates the shifting landscape of cancer treatment, showing a transition from traditional chemotherapy to more targeted and immune-based approaches. Chemotherapy Agents exhibit the strongest relationships with Phytogenic Agents, Supportive Therapies, and Targeted Therapies, reflecting their central role in both established and evolving regimens. Moderate connections between Monoclonal Antibodies, Immune Checkpoints, and Targeted Therapies indicate growing interest in immunotherapy. Weaker links, such as those involving Tumor Suppressors, suggest more specialized research focus. Relationship strengths between categories are quantified in the **category_pairs.tsv** file. Emerging patterns highlight the increasing integration of biomarkers and growth factor receptors, and the expanding but still developing role of immunotherapies. Across all categories, **Supportive Care** remains vital for managing side effects and enhancing treatment tolerability. Figure 7 provides an overview of these trends based on *NameOfSubstance* trigram frequencies.

### 3.4. COVID-19 Pandemic Impact on Research Directions

To assess the impact of the COVID-19 pandemic on scientific publications, we compared the observed data to a baseline representing expected publications in a normal year (Appendix A.4). Key trends include the following:

- 2020 (Surge +24.64%): Publications increased sharply to 3197 articles, with a deviation of +335.9%, indicating a major shift in research activity.
- 2021 (Peak +8.20%): Publications rose to 3459 articles, reaching a maximum deviation of +534.8%, marking the peak of pandemic-driven research.
- 2022–2023 (Plateau 0.37–0.72%): Growth slowed but remained high, with a deviation above +375%, indicating a new steady-state.
- 2024 (Stabilization −0.97%): Publications slightly declined to 3463, but still remained above historical trends, suggesting a structural shift.

**Figure 8:**
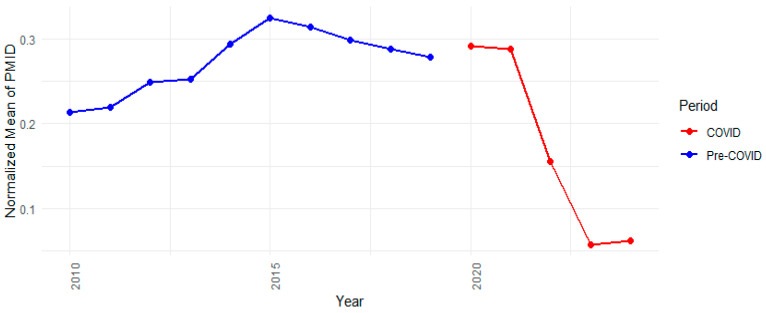
Trigrams and deviations trend during COVID-19 pandemic.

The COVID-19 pandemic significantly impacted the quantity of scientific publications. To assess its effect on anti-cancer drug research, we analyzed trigrams trends. We calculated the annual average PMID counts for relevant trigrams, normalized the data based on each trigram’s maximum occurrence, and plotted these trends to visualize shifts in research focus over time (Figure 8). The normalized values represent the average proportional mention intensity of trigrams across PubMed articles from 2010 onward.

**Figure 9:**
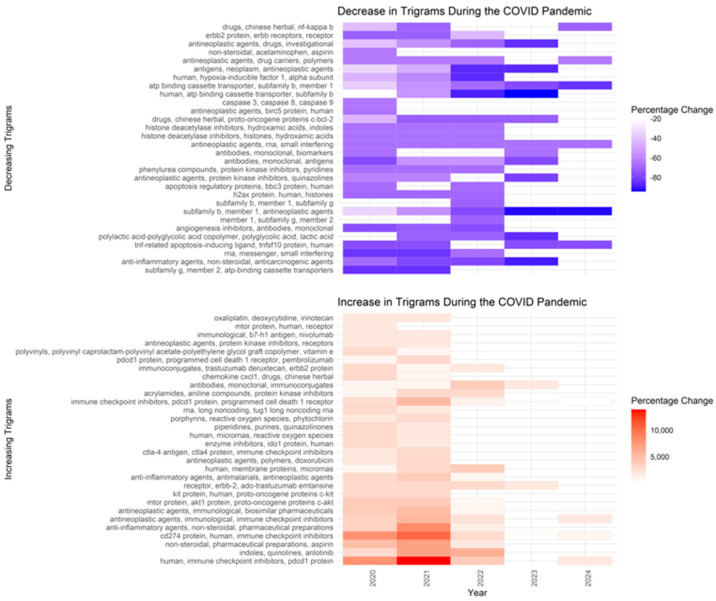
Heatmaps illustrating the percentage change in *NameOfSubstance* trigram counts during the COVID-19 pandemic wave. The top panel shows the 30 most decreased trigrams, while the bottom panel highlights the 30 most increased.

From 2010 to 2019, there was a steady increase, peaking at 0.324 in 2015. During the pandemic (2020–2024), there was a brief rebound to 0.292 in 2020, likely due to oncology studies incorporating pandemic-related topics like immunosuppression, drug repurposing, and antiviral synergy. However, this was followed by a decline (0.155 in 2022 and approximately 0.06 by 2023–2024). Possible reasons include the reallocation of research due to pandemic restrictions, a shift toward mRNA therapeutics and vaccines, and funding changes that deprioritized certain areas [18,19,20]. Additionally, *NameOfSubstance* trigrams were used to assess shifts in specific research areas (Figure 9).

The datasets show a surge in references to mRNA- and RNA-based platforms, such as “mRNA vaccines” and “RNA-based therapies”, driven by the success of COVID-19 vaccines [21]. Growth in trigrams like “ICIs” and “CAR T-cells” reflects rising interest in advanced immuno-oncology [22,23], while increased mentions of supportive and palliative care relate to heightened awareness of complications in immunocompromised patients [24]. Short-term spikes were observed for repurposed COVID-19 drugs, including tocilizumab and baricitinib. Conversely, mentions of older chemotherapy terms like “alkylating agents” and “antimetabolites” declined, reflecting a shift toward immunotherapies and targeted treatments [2]. The pandemic also slowed niche research due to lab shutdowns and redirected resources [19], and Phase III trials faced enrollment challenges, lowering citation rates for complex regimens [6].

**Figure A2:**
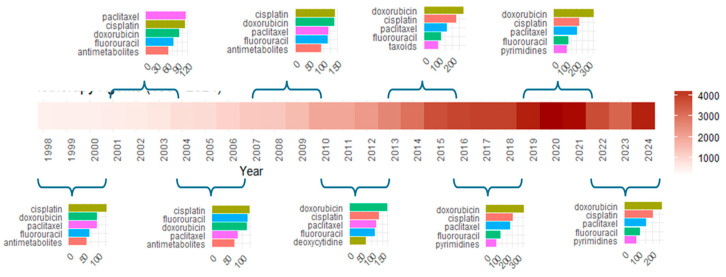
The central heatmap represents the absolute PMID count per year, with darker shades indicating higher research activity. The surrounding bar charts highlight the top five most frequently mentioned chemotherapy agents at different time intervals, illustrating shifts in research focus.

Looking ahead, big data analytics and real-world evidence are expected to reshape treatment strategies and influence publication trends [16,24]. As detailed in Appendix A.5, three key areas stand out in anti-cancer drug research: Chemotherapy Agents, for their foundational role; Immune Checkpoints, for their transformative therapeutic impact; and Vaccines, particularly those accelerated by the COVID-19 response [21,22,23]. While newer modalities gain traction, cytotoxic chemotherapy remains integral, with ongoing refinements in dosing, scheduling, and combinations with biologics (Appendix A.5 Figure A2).

### 3.5. Publication Trends

#### 3.5.1. Immune Checkpoint

**Figure 10:**
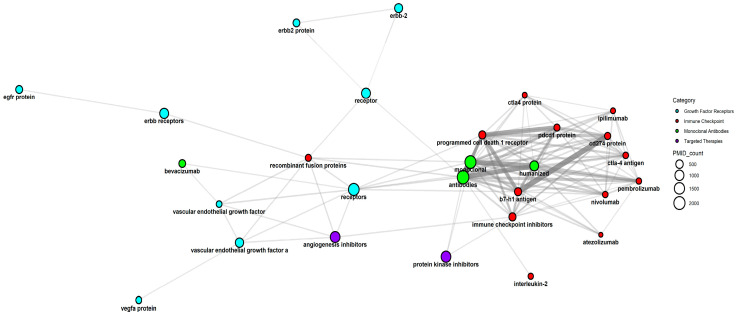
Relationships among Immune Checkpoint-related substances. Nodes represent different substances, with size proportional to their PMID count. Edges indicate co-occurrence within the same study. Stronger connections (darker edges) highlight frequent co-citations, suggesting stronger associations in the research literature. Categories are color coded as shown in the right-side legend.

The Immune Checkpoint category is central in cancer therapy, connecting with various other categories such as monoclonal antibodies, targeted therapies, and traditional chemotherapy. Its effectiveness is influenced by factors like inflammation, drug resistance, and tumor suppressor gene status (Figure 10). Understanding these intricate relationships allows for more strategic combination therapies and better-informed clinical decisions.

An overview of how the Immune Checkpoint category functionally and therapeutically interacts with other major categories is shown in Table 3, based on the classification data (file classification_NameOfSubstance.txt for NameOfSubstance classification) and the output from graph generation (ImmuneCheckpoint_filtered_immune_data_with_titles.tsv) in the Supplementary Materials, alongside established scientific evidence in oncology and immunology.

A total of 243 articles referencing ICIs in the *NameOfSubstance* field reveal evolving trends in immunotherapy, including safety, biomarker-driven personalization, and combination strategies. Early focus on PD-1/PD-L1 and CTLA-4 has expanded to include LAG-3, TIGIT, and PVRIG, reflecting broadening checkpoint targets. From 2019 to 2025, ICI applications have grown across both immunogenic cancers (e.g., melanoma) and traditionally resistant types like KRAS-mutant pancreatic cancer and microsatellite-stable colorectal cancer, with synergistic effects observed in non-small cell lung cancer (NSCLC) treated with EGFR inhibitors and ICIs [25,26].

Recent studies emphasize combinatorial approaches, pairing ICIs with tyrosine kinase inhibitors, anti-angiogenics, and epigenetic modulators (e.g., HDAC and DNA methylation inhibitors) to overcome resistance and improve efficacy [27,28,29,30,31,32]. Persistent efforts focus on resistant tumors such as MSS colorectal cancer and KRAS-driven malignancies [33,34]. Innovative drug delivery systems, like nanocomposite gels and liposomal carriers, aim to localize immune effects and reduce systemic toxicity [35,36].

Classic drugs—cisplatin, paclitaxel, sorafenib, trastuzumab, and others—remain central, often featured in combination trials, underscoring the role of immunomodulatory synergy between traditional and immune-based treatments. The presence of analgesics, antacids, and metabolic modulators suggests an emerging interest in how supportive medications influence the tumor immune microenvironment. Lower-frequency mentions in Q3/Q4 may represent early-stage agents or niche approaches with future clinical potentials.

#### 3.5.2. Cancer Vaccine Therapies

Cancer vaccines are rapidly emerging as a pivotal area in immuno-oncology, aimed at providing durable antitumor immunity. These vaccines, which can complement existing therapies like ICIs, targeted therapies, and chemotherapy offer significant promise for advancing cancer treatment strategies. A filtered dataset of 306 cancer vaccine-related records (detailed in vaccine_data.tsv in the Supplementary Materials) provides valuable insights into this evolving field.

The dataset distinguishes between Prophylactic Vaccines, designed to prevent infection-related cancers (e.g., HPV vaccines for cervical, anal, and oropharyngeal cancers [10] and HBV vaccines to reduce liver cancer risk [9]), and therapeutic vaccines, aimed at treating existing cancers by enhancing the immune response to tumor antigens [4,11,12]. ICIs, like anti-PD-1 and anti-CTLA-4, complement therapeutic vaccines by expanding tumor-specific T-cell populations and preventing immune exhaustion [23]. Chemotherapy can enhance vaccine efficacy through immunogenic cell death, which releases tumor antigens and boosts immune responses, while radiotherapy further modulates the tumor microenvironment [37]. Targeted therapies, such as EGFR or VEGF blockade, also aid in vaccine effectiveness by increasing antigen release and immune infiltration.

Emerging platforms like mRNA-based vaccines and dendritic cell vaccines (as noted in vaccine_data.tsv) are gaining traction, with early-phase clinical trials showing promising immune responses. Oncolytic virus-based vaccines, like T-VEC, function as virotherapy while enhancing antitumor immunity through tumor antigen expression [13].

The dataset also highlights three principal clinical challenges: tumor heterogeneity, which requires personalized or multi-antigen approaches [14]; immunosuppression, where combining vaccines with ICIs or immunomodulatory agents is crucial; and limited immunogenicity of self-derived tumor antigens, necessitating careful adjuvant and epitope selection [38]. The data reflect an expanding pipeline of neoantigen vaccines, dendritic cell-based vaccines, and next-generation RNA vaccines, with therapeutic vaccines continuing extensive clinical testing.

## 4. Discussion

Our comprehensive bibliometric and content-based assessment of PubMed records highlight the swift evolution of anti-cancer drug research over multiple decades.

While we have meticulously employed rigorous bibliometric methodologies to mitigate potential biases, it is important to acknowledge inherent limitations. Specifically, certain influential factors and phenomena remain elusive in purely bibliometric analyses of PubMed records. Although institutional affiliations such as universities, institutes, or foundations, as well as broad categories of funding sources, are explicitly identified during the manuscript submission process, numerous underlying drivers of research trends may remain hidden. Factors including parallel financing streams, opportunistic research initiatives by individual researchers or departments, and other transient or emergent influences could significantly impact research trajectories. Capturing these dynamics would necessitate alternative research methodologies and a distinct analytical focus oriented towards identifying and characterizing such “driving forces,” rather than solely quantifying trends from bibliometric datasets.

The analysis revealed a pronounced acceleration in research output during key inflection points, such as the onset of the COVD-19 pandemic. The exponential growth of publications reflects both technological and clinical advances, including the advent of high-throughput genomic platforms and the surge in immuno-oncology. In particular, our classification scheme, which organizes substances into categories like chemotherapy, immune checkpoint, targeted therapies, and phytogenic agents, highlights the expanding complexity of contemporary oncology. The results also confirm the ongoing importance of older cytotoxic regimens in resource-limited settings and as backbones for combination protocols.

Several findings stand out. First, classical chemotherapy agents, despite the rise in targeted and immune-based strategies, remain fundamental to many clinical protocols, illustrating the durability of well-established cytotoxic mechanisms. Second, immunotherapies have reshaped treatment paradigms with notable success in various tumor types, yet their broad adoption also raises issues of drug resistance, long-term toxicities, and heterogeneous response rates. Third, the persistent integration of biomarkers—circulating RNA, protein markers, and genomic variants—signals a collective effort to refine patient stratification and optimize therapeutic outcomes. Finally, supportive therapies addressing side effects and comorbidities emerge as crucial in extending survival and enhancing patient quality of life, especially in multi-drug combination regimens.

In the context of prior research, these trends mirror the widely acknowledged shift towards personalized medicine: we observe greater reliance on combination strategies that merge targeted agents, immunomodulators, and classical drugs, with robust supportive care to manage adverse events. This integrative paradigm aims to exploit multiple vulnerabilities within tumors while improving tolerability. The data further suggest that emerging areas, such as mRNA-based vaccines and advanced nanocarrier delivery systems, could drive the next wave of innovation, accelerating synergy with immunotherapies and enabling more precise drug deployment.

Looking forward, future research directions may revolve around three core themes: (1) deepening multi-omics profiling to better predict therapy responses and tailor regimens to individual tumor biology; (2) developing improved mechanisms to overcome or preempt drug resistance—particularly by targeting ABC transporters, modulating tumor microenvironments, or leveraging synthetic lethality approaches; and (3) adopting advanced data-driven methodologies, including real-world evidence and machine learning, to more rapidly optimize treatment sequencing and dosage. Taken together, these converging lines of inquiry are poised to redefine anti-cancer drug development, promoting a multifaceted and patient-centered approach that aligns discovery with evolving clinical needs.

Bibliometric data show a pronounced shift toward personalized cancer medicine, evidenced by the frequent co-occurrence of ICIs, and advanced drug delivery platforms such as nanocarriers and immunoconjugates. At the same time, high publication counts on supportive care therapies highlight an ongoing commitment to optimizing patient outcomes through toxicity mitigation and quality-of-life measures. Our classification scheme, based on the *NameOfSubstance* field, underscores the complex interplay among drug resistance mechanisms, growth factor signaling pathways, immunomodulation, and emerging vaccine technologies. Taken together, these findings indicate that future research will likely focus on synergy-oriented regimens—integrating established cytotoxins, new molecular targets, immune-based strategies, and innovative formulations.

The rise in biotechnology has led to increased investment in research and clinical trials involving monoclonal antibodies. In this context, a range of combinatorial strategies has been employed to strengthen the clinical evidence supporting ICIs and to promote their potential use in refractory cancers such as pancreatic cancer, as demonstrated both in this study and in previously published research [39,40,41].

## 5. Conclusions

By systematically mapping past and present trends, this study provides a foundation for anticipating how forthcoming scientific advances, global health events, and collaborative consortia will continue to reshape the anti-cancer drug landscape. Capturing these recurring trigrams across such diverse categories, we see a richly interlinked oncology field, where formerly siloed approaches—like chemotherapy, immunotherapy, and supportive therapies—converge into strategic, patient-centric regimens. This integration reflects a paradigm shift in cancer treatment, emphasizing a holistic approach where different therapeutic modalities are no longer viewed in isolation but rather as complementary components of a broader, more effective strategy. Future research will likely build on this convergence, leveraging multi-modal interventions that optimize treatment efficacy while minimizing adverse effects, ultimately driving improvements in patient survival and quality of life.

## Figures and Tables

**Table 1:** Descriptive statistics of number of scientific publications over time related to “*anti-cancer drugs*” PubMed prompt.

**min**	**MAX**	**mean**	**median**	**SD**	**variance**
1	3497	733.5079	245	1037.403	1,076,204
**Q1**	**Q3**	**IQR**	**range**	**skewness**	**kurtosis**
15	948.5	933.5	3496	1.515994	1.13817

**Table 2:** Overall statistics of PubMed records related to “*anti-cancer drugs*” PubMed prompt, analyzing distribution of PMID fields *NameOfSubstance*, *DescriptorName*, and *Keywords*.

	NameOf Substance	Descriptor Name	Keywords
TotalPMIDs	37,521	39,237	23,330
Total Unique Elements	14,456	13,105	47,107
Median Elements Per PMID	5	14	5
Max Elements	39	54	216
PMIDs With 1 Element	3280	5	17
PMIDs With 2 Elements	4030	26	88
PMIDs With 3 Elements	4796	118	1066
PMIDs With > 3 Elements	25,415	39,088	22,159

**Table 3:** Table summarizing some examples of combinations of ICIs with each category and brief explanation of rationale.

Category	Example of Combination with ICIs	Rationale
Drug Resistance	ATP-binding cassette transporters	Chronic immune evasion can coincide with increased expression of drug-resistance genes. Tumors that acquire resistance to classical agents may still respond to immunotherapies targeting the PD-1/PD-L1 or CTLA-4 pathways
Anti-inflammatory	COX-2 inhibitor	Anti-inflammatory milieu might enhance or occasionally reduce the efficacy of checkpoint inhibitors
Phytogenic agents/ Chinese Herbal	flavonoids, terpenoids	Enhance dendritic cell function or modulate T regulatory cells
Growth Factor Receptors	EGFR, VEGF/VEGFR	Normalize the tumor vasculature and improve immune cell access
Targeted Therapies	BRAF, EGFR, or mTOR inhibitors	Boost tumor antigen release and create a favorable setting for T-cell priming
Chemotherapy Agents	chemotherapy	Chemotherapy-induced cell death can generate antigenic debris, prompting dendritic cell activation, ICIs support T-cell-mediated clearance of residual tumor cells
Tumor Suppressors	BCL-2, BAX, TP53	Combined modulation of cell death pathways and immune checkpoints can lead to robust tumor regression as consequence of Fas ligand and TNF-related apoptosis
Nucleic Acids/ Gene Therapies	siRNA, mRNA vaccines	Enhance T-cell-mediated killing
Drug Delivery/ Advanced Formulations	liposomes, nanoparticles	Improved release of drugs and off-target effects

**Figure A1:**
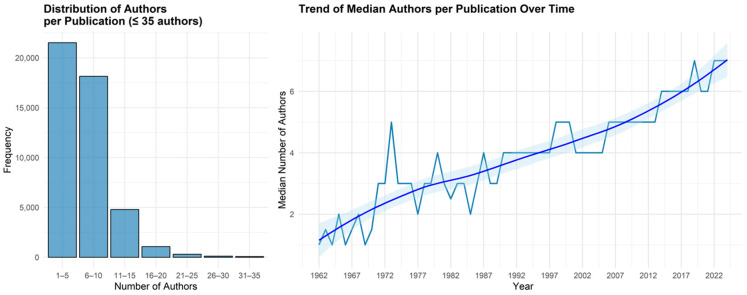
Distribution and trend of authors per publication. Left Panel: Distribution of the number of authors per publication, grouped into bins of five authors (cutoff 35th author). Right Panel: Time series plot displaying the trend of the median number of authors per publication from the earliest available data to 2024. The blue line represents the yearly median number of authors, while the smoothed LOESS curve (light blue shaded area) highlights the overall increasing trend over time.

## Data Availability

The data presented in this study are available in PubMed at https://pubmed.ncbi.nlm.nih.gov/. These data were acquired through PubMed Central (PMC) APIs. The most informative derived datasets obtained through queries of the original downloaded dataset are uploaded in the Supplementary Materials.

## Supplementary Materials

The following supporting information can be downloaded at: https://www.mdpi.com/article/10.3390/pharmaceutics17050610/s1.

## Appendix A

### Appendix A.1. Classification, Key Observations, and Notable Substances

Apoptosis and Cell Death:

- **Reactive Oxygen Species (ROS)** play a role in inducing oxidative stress and apoptosis in cancer cells.
- **Caspases** (e.g., caspase 3, 8, 9) are key executors of apoptosis.
- **BCL Family Proteins** regulate mitochondrial apoptosis pathways. Proteins like **BAX** promote apoptosis, while **BCL2** and **BCL-X** can block it.
- **Fas/FasL** are involved in the extrinsic apoptosis pathway.
- **Inhibitors of Apoptosis (IAP)** proteins help tumor cells evade apoptosis.
- **TNF and TNF-Alpha** cytokines can induce cell death or survival depending on context.
- **Survivin (BIRC5)** and **Mcl-1** are overexpressed in cancers to prevent apoptosis.
- **NF-κB** regulates apoptosis, often associated with inflammation and cancer survival.
- **HIF1A** and **Hypoxia-Inducible Factor 1** influence apoptosis under hypoxic conditions in tumors.
- **Heat-shock proteins (HSP70, HSP90)** protect cells from stress-induced apoptosis.
- **c-Myc** and **MDM2** modulate apoptosis, with MDM2 negatively regulating p53.

**Insights and Hypothesis**: The high count for NF-κB (668) suggests interest in modulating NF-κB pathways to influence cell survival or death. A promising therapeutic approach could be co-targeting NF-κB and caspase pathways in resistant tumors. The focus on TNF-α over TNF points to targeting TNF-α specific signaling for apoptosis induction or tumor progression reduction. Bcl-x’s higher count than Bcl2 raises interest in targeting Bcl-x in anti-apoptotic strategies.

Chemotherapy Agents:

- **DNA-Damaging Agents** include alkylating agents, organoplatinum compounds like **cisplatin**, **oxaliplatin**, and **carboplatin**, along with **fluorouracil**, **methotrexate**, **gemcitabine**, **capecitabine**, and **cytarabine**.
- **Topoisomerase Inhibitors** such as **etoposide**, **doxorubicin**, **camptothecin**, **irinotecan**, and **topotecan** target cancer DNA.
- **Microtubule-Targeting Drugs** like **paclitaxel**, **docetaxel**, **vincristine**, and **vinblastine** inhibit cell division.
- **Others** include **bleomycin**, **mitomycin**, **mitoxantrone**, and **arsenic trioxide**.

**Insights and Hypothesis**: Doxorubicin (1787) continues to be heavily researched, indicating persistent challenges with its cardiotoxicity. Future studies may focus on liposomal doxorubicin to reduce toxicity. The higher mention of cisplatin (1483) compared **to** oxaliplatin (331) reflects cisplatin’s historical prominence, although future research might favor oxaliplatin or newer platinum analogs with improved safety.

Growth Factor Receptors:

- **ERBB receptors** (951), including **EGFR** and **HER2/ErbB2**, are involved in breast, lung, and other cancers.
- **EGF/VEGFR** play critical roles in angiogenesis.
- **IGF** and **FGF** families are also implicated in tumor growth.

**Insights and Hypothesis**: Targeted therapies against HER2/ErbB2 (e.g., trastuzumab, pertuzumab) remain crucial in clinical settings. VEGF/VEGFR continues to be a focus, and future research may combine anti-VEGF therapies with immunotherapy to combat resistance and improve cancer treatment outcomes.

Immune Checkpoint:

- **Immune checkpoint inhibitors (ICIs)** target **PD-1**, **PD-L1**, and **CTLA-4**.
- **PD-1/PD-L1 pathway** includes **PD-1, PD-L1, Nivolumab, Pembrolizumab,** and **Atezolizumab.**
- **CTLA-4 pathway** includes **Ipilimumab**.
- **Recombinant Fusion Proteins** are being explored for blocking immune checkpoints.

**Insights and Hypothesis**: Research on PD-1/PD-L1 inhibitors continues to boom, suggesting potential for combination therapies like PD-1 + CTLA-4. The growing interest in Recombinant Fusion Proteins indicates further exploration of checkpoint receptors beyond PD-1 and CTLA-4.

Anti-inflammatory Drugs:

- **NSAIDs**: Common non-steroidal anti-inflammatory drugs like **aspirin**, **ibuprofen**, and **celecoxib**.
- **COX Enzymes**: **COX-2** and **COX-1** are targets for inflammation control.
- **Prostaglandins** and **Arachidonic Acid** are involved in inflammatory pathways.
- **Interleukin-2** also plays a role in certain immunotherapies.

**Insights and Hypothesis**: The high count of NSAIDs (2525) reflects their broad application. The focus on celecoxib (249) suggests the potential of COX-2 inhibitors in combination with chemotherapy. Ongoing trials on low-dose aspirin for cancer prevention may reduce tumor incidences.

Monoclonal Antibodies:

- **Trastuzumab** and **bevacizumab** are key in targeting **HER2** and **VEGF**, respectively.
- **Cetuximab**, **rituximab**, and **panitumumab** target **EGFR** and **CD20**.
- **Bispecific antibodies** and **immunoconjugates** are newer research directions.

**Insights and Hypothesis**: The predominance of humanized antibodies suggests a focus on reducing immunogenicity. Immunoconjugates are a rising area, likely due to success stories like T-DM1 in breast cancer.

Cytokines:

- **Interferons** (e.g., **IFN-γ**, **IFN-α**) help boost immune responses against tumors.
- **Granulocyte Colony-Stimulating Factor** and **GM-CSF** support immune cell activity.
- **Cancer vaccines** aim to elicit immune responses against tumor antigens.

**Insights and Hypothesis**: Research is shifting towards combination therapies using checkpoint inhibitors and adoptive cell therapies. Cancer vaccines focused on tumor antigens are also gaining traction.

Supportive Therapies:

- **Opioids**, **Analgesics**, **Antiemetics**: Manage pain, nausea, and other side effects in cancer patients.
- **Diphosphonates** help manage bone metastases.

**Insights and Hypothesis**: Pain management remains critical in oncology. As immunotherapies rise, the complexity of immune-related adverse events will likely necessitate further supportive care research.

Phytogenic Agents:

- **Chinese herbal** (1638) and **plant extracts** (895) are extensively researched for potential anti-cancer properties.
- **Curcumin**, **Resveratrol**, and **Quercetin** are among the most studied compounds.

**Insights and Hypothesis**: The significant interest in Chinese herbal remedies reflects growing interest in Traditional Chinese Medicine (TCM). Future studies may focus on combining phytogenic agents with Western therapeutics to enhance cancer treatment outcomes.

Targeted Therapies:

- Protein Kinase Inhibitors (1305) and Enzyme Inhibitors (930) remain a focus.
- PARP Inhibitors and Proteasome Inhibitors (e.g., Bortezomib) are also prominent.

**Insights and Hypothesis**: Protein Kinase Inhibitors remain a primary research focus. PARP inhibitors are gaining attention for targeting BRCA-mutated tumors, indicating the growing interest in synthetic lethality approaches.

Tumor Suppressors:

- **p53** is the most studied tumor suppressor, with **p21** and **p27** playing key roles in cell cycle regulation.

**Insights and Hypothesis**: Restoring p53 function could be transformative for refractory cancers, especially through MDM2-p53 inhibitors. Targeting p21 may offer a promising approach to halt tumor progression.

### Appendix A.2. Shifting Patterns in Scientific Collaboration: Trends in Authorship Count

The analysis of authorship trends in scientific publications, shown in Figure A1 and Table A1, reveals a growing trend of large-scale collaborations in research. The left panel shows that most publications are still produced by small- to mid-sized teams (1–10 authors), but there are more publications with higher author counts, indicating larger collaborations. The right panel shows a steady increase in the median number of authors per publication, especially after 2010. The median has risen to 7–8 authors in 2020–2024, reflecting the increasing need for interdisciplinary teams. Extreme cases, like a publication with 2440 authors in 2016, highlight the rise in consortia-driven studies, particularly in fields like genomics and clinical trials. In 2023, another article had 228 authors, demonstrating the trend of large collaborations. This shift towards larger teams reflects the growing complexity of modern research, especially in areas like big data, artificial intelligence, and global health. These outliers show how large consortia play a key role in addressing complex scientific challenges. As research demands continue to increase, larger, interdisciplinary teams will likely remain essential.

**Table A1:** Summary statistics on number of authors per scientific publication over time.

Year	N. Articles	Mean Authors	Median Authors	Min Authors	Max Authors	Q1	Q3
2000	459	5	5	1	24	2	7
2001	505	5	4	1	21	2	6
2002	661	5	4	1	40	2	7
2003	662	5	4	1	24	2	6
2004	706	5	4	1	25	3	7
2005	818	5	4	1	33	3	7
2006	839	5	5	1	25	3	7
2007	830	5	5	1	25	3	7
2008	880	5	5	1	28	3	7
2009	1016	6	5	1	23	3	8
2010	1182	6	5	1	24	3	8
2011	1216	6	5	1	26	3	8
2012	1333	6	5	1	21	3	8
2013	1468	6	5	1	34	3	8
2014	1752	6	6	1	35	4	9
2015	1974	7	6	1	71	4	9
2016	2111	9	6	1	2440	4	9
2017	2144	7	6	1	40	4	9
2018	2425	7	6	1	38	4	9
2019	2564	7	7	1	44	4	9
2020	3190	7	6	1	57	4	9
2021	3453	7	6	1	76	4	9
2022	3471	8	7	1	80	4	10
2023	3490	8	7	1	228	5	10
2024	3449	8	7	1	112	5	10

### Appendix A.3. Anti-Cancer Drug Classification Insights

**Apoptosis and Cell Death:**

Research into apoptosis grew significantly from the late 1990s, peaking at 1.0 in 2022. This reflects the growing importance of apoptosis in cancer therapy. Potential Hypothesis: Future therapies may combine apoptotic regulators (e.g., Bcl-2 inhibitors) with immunotherapies to improve cancer cell clearance and reduce drug resistance.

**Chemotherapy Agents:**

Chemotherapy remained a strong research focus, peaking in 2021 at 1.0. The growth reflects improvements in formulations, combinations, and side effect mitigation. Potential Hypothesis: Next-generation chemotherapies will be combined with immunotherapies and targeted agents to improve efficacy and reduce toxicities.

**Growth Factor Receptors:**

Publications rose sharply after 2000, peaking in 2021 at 1.0. This mirrors the success of therapies targeting EGFR and HER2, and anti-angiogenic strategies. Potential Hypothesis: Future therapies may target multiple receptors or use bispecific antibodies to prevent tumor escape.

**Immune Checkpoint:**

After minimal interest in the early 2000s, research surged after 2010, with a peak in 2021. PD-1/PD-L1 and CTLA-4 inhibitors have revolutionized cancer treatment. Potential Hypothesis: Combinations of checkpoint inhibitors or new targets (e.g., LAG-3, TIM-3) could broaden effectiveness.

**Anti-inflammatory Drugs:**

Research began in the 1990s but gained momentum around 2005–2010, peaking at 1.0 in 2021. This highlights the role of chronic inflammation in tumorigenesis. Potential Hypothesis: Combining anti-inflammatory strategies with immunotherapy could enhance immune response and limit tumor-promoting inflammation.

**Monoclonal Antibodies:**

Starting with minimal research, this area grew steadily, peaking at 1.0 in 2021. The rise follows FDA approvals of trastuzumab, bevacizumab, and rituximab. Potential Hypothesis: Next-generation antibodies, like T-cell engagers and bispecific, will improve tumor specificity and reduce toxicity.

**Phytogenic Agents:**

Research into natural compounds grew steadily, peaking at 1.0 in 2021. Interest in plant-derived compounds with anti-cancer properties remains strong. Potential Hypothesis: Continued screening and standardization of these compounds could lead to new treatments that complement traditional therapies.

**Supportive Therapies:**

Publications on palliative and symptomatic care increased steadily, reaching a peak at 1.0 in 2021.

Potential Hypothesis: Future supportive care may integrate digital tools, personalized symptom monitoring, and proactive measures to reduce treatment-related toxicities.

**Targeted Therapies:**

Interest grew significantly from the late 1990s, peaking in 2021 at 1.0. This growth is driven by advances in tyrosine kinase inhibitors and other targeted agents. Potential Hypothesis: New inhibitors will target protein–protein interactions, epigenetic changes, and allosteric sites to overcome resistance.

**Tumor Suppressors:**

Interest in tumor suppressors grew steadily after 2000, peaking at 1.0 in 2017. Research focuses on restoring the function of genes like TP53, RB1, and PTEN. Potential Hypothesis: Strategies to restore p53 function or reactivate epigenetically silenced suppressors could enhance immunotherapy and targeted treatments.

### Appendix A.4. COVID-19 Pandemic Impact on Scientific Publication

A linear regression model (N ∼ Year) was fitted to the historical data (pre-2019) to illustrate the long-term growth trend in publications. This model was then used to predict expected publication values for 2020–2024, forming a dynamic baseline (*T**r**e**n**d*). To reduce short-term fluctuations, a 10-year rolling average (k = 10) was computed, enhancing the reliability of the baseline estimate. The baseline for 2020–2024 was linearly interpolated between the observed values for 2019 and 2024, providing a continuous reference curve for comparison with actual publication counts.

Once the baseline was established, deviations were assessed using the following metrics:

- **Absolute Deviation from Baseline (SpikeHeight)**: The difference between the observed publications and the baseline in a given year.

*SpikeHeight* = *N* − *Baseline* (A1)

- **Percent Deviation from Baseline (PercentVariation)**: The deviation as a percentage relative to the historical average.

PercentVariation = N − μ × 100 (A2)

where μ\muμ is the historical average publication count.

- **Outlier Detection via Standard Deviation**: A year was classified as a statistical anomaly if it exceeded μ + 2σ\mu + 2\sigmaμ + 2σ, where σ\sigmaσ is the standard deviation of the historical data.

*μ* + 2*σ* (A3)

- **Peak Area Calculation**: The area under the curve between the observed publication count and the baseline was computed to quantify the total excess publications during the pandemic period.

Area = N − Baseline (A4)

- **Identification of the Maximum Peak**: The year with the highest deviation was extracted, marking the peak of the increase in scientific output.

Interestingly, the largest deviation from the baseline occurred in 2021, with an excess of 534.8 publications, while total area under the excess curve from 2020 to 2024 amounted to 1581 publications above the expected trend (Table A2).

These calculations revealed a significant surge in publications during the pandemic period, particularly in 2021, highlighting the increased focus on urgent scientific topics like COVID-19 and related research.

**Table A2:** Descriptive statistics of number of “*anti-cancer drugs*” PubMed prompt.

Year	Publications (N)	Percent Change (Δ%)	Deviation from Baseline	Spike Height (N in Excess Compared to Baseline)	Peak Area (Cumulative N in Excess Compared to Baseline)
2019	2565	5.64%	+249.7%	0	0
2020	3197	24.64%	+335.9%	452	452
2021	3459	8.20%	+371.6%	534	987
2022	3484	0.72%	+375.0%	380	1367
2023	3497	0.37%	+376.8%	213	1581
2024	3463	−0.97%	+372.1%	0.0	1581

### Appendix A.5. Substance-Related Trigrams and Research Trends

**Apoptosis and Cell Death:**

Early research focused on **caspase proteins**, **bcl2**, and **TNF** in apoptosis. Over time, trigrams involving **p53**, **mdm2**, and **ROS** gained prominence, reflecting a deeper understanding of both intrinsic and extrinsic apoptotic pathways. **Trend**: Publications surged in the late 1990s–2000s, aligned with the development of pro-apoptotic therapeutics (e.g., **BH3 mimetics**). **Outlook**: Future research may focus on **Bcl-2 inhibitors** and **mdm2-p53 disruptors** combined with immunotherapies to reduce resistance.

**Chemotherapy Agents:**

Despite the rise in targeted therapies, chemotherapy terms like **doxorubicin**, **fluorouracil**, and **cisplatin** remain highly cited. **Trend**: Peaks in the 1990s–2000s reflected the establishment of chemotherapy as a backbone in cancer treatment. **Outlook**: **Nanocarriers** and **chemoimmunotherapy** will ensure chemotherapy remains vital, especially in resource-limited settings.

**Growth Factor Receptors:**

Keywords related to **EGFR**, **HER2**, and **VEGF** surged in the 2000s as targeted therapies (e.g., **gefitinib**, **trastuzumab**) became standard. **Trend**: Research continues to expand, particularly in **NSCLC**, **breast**, and **colorectal cancers**. **Outlook**: Combining receptor inhibitors with immunotherapy will remain a key focus.

**Immune Checkpoint:**

Emerging terms like **PD-1**, **CTLA-4**, and **immune checkpoint inhibitors** (e.g., **nivolumab**, **pembrolizumab**) have dominated research since 2010. **Trend**: Immune checkpoint inhibitors have become crucial in treating solid tumors. **Outlook**: Future studies will focus on next-gen checkpoints (e.g., **LAG-3**, **TIGIT**) and combination therapies.

**Cytokines:**

Trigrams involving **interferons**, **interleukins**, and **cancer vaccines** have remained present. **Trend**: Cytokine therapies and vaccines continue to play a role in immunotherapy, often in combination with checkpoint inhibitors. **Outlook**: Personalized cancer vaccines and engineered T-cell approaches will drive the next wave of immunotherapies.

**Anti-inflammatory Drugs:**

Keywords like **COX-2 inhibitors** (e.g., **celecoxib**) and **aspirin** continue to feature prominently, reflecting interest in inflammation’s role in cancer. **Trend**: NSAIDs and COX inhibitors gained attention in the 2000s, linking aspirin use with cancer prevention. **Outlook**: Future research may explore **aspirin + checkpoint blockade** or **NSAIDs + immunotherapy** combinations.

**Monoclonal Antibodies:**

Monoclonal antibodies like **trastuzumab**, **bevacizumab**, and **rituximab** have seen steady growth. **Trend**: Monoclonal antibodies revolutionized oncology from the late 1990s. **Outlook**: Advanced formats like **antibody-drug conjugates** and **T-cell engagers** will further expand the field.

**Phytogenic Agents:**

Research into plant-derived compounds, including **flavonoids** and **alkaloids**, has grown, with increasing mentions of **Chinese herbal medicine**. **Trend**: The use of herbal compounds has expanded, particularly in complementary oncology. **Outlook**: Combination with modern therapies (e.g., **phytochemicals + checkpoint blockade**) could integrate phytogenic agents into oncology.

**Supportive Therapies:**

Trigrams related to **pain management**, **antiemetics**, and **infection prophylaxis** have grown, reflecting the need for symptom control in oncology. **Trend**: Supportive care has become more integral, reflecting the rise in combination regimens and longer patient survival. **Outlook**: Digital health tools and prophylactic interventions will play a larger role in supportive therapies.

**Targeted Therapies:**

Research on **tyrosine kinase inhibitors**, **BRAF**, and **PARP inhibitors** has dominated since the early 2000s. **Trend**: Targeted therapies have become central in modern oncology. **Outlook**: Combination strategies (e.g., **PARP inhibitors + PD-L1 blockade**) and novel targeting paradigms (e.g., **allosteric modulators**) will address resistance.

**Tumor Suppressors:**

References to **p53**, **CDKN1A**, and **PTEN** have highlighted efforts to restore tumor suppressor functions. **Trend**: Research has increased since the 1990s, focusing on the molecular mechanisms of cancer. **Outlook**: Reactivating tumor suppressors (e.g., **p53** via **mdm2-p53 blockers**) will complement targeted therapies and immunotherapies.

A trend across all categories is the focus on **combination therapies**. Classic chemotherapies, targeted therapies, immunotherapy, and supportive treatments are increasingly integrated into comprehensive treatment frameworks.

**Key Areas for the Future:**

- **Synergistic Therapies**: Combining apoptosis-inducing strategies with checkpoint blockers or growth factor receptor inhibitors.
- **Personalized Biomarkers**: Multi-omics panels to enable the precise use of treatments and monitor minimal residual disease.
- **Advanced Formulations**: Liposomal- or nanoparticle-based systems for targeted delivery.
- **Expanded Immuno-Oncology**: Incorporating next-gen immune checkpoints, adoptive T-cell therapies, and vaccine-based approaches.
- **Phytogenic and Integrative Treatments**: Integrating plant-derived agents with modern therapies.
